# First Bisexually Dimorphic Phytoptid Taxon (Eriophyoidea, Phytoptidae) from Gondwanian Angiosperm Host

**DOI:** 10.3390/ani15091236

**Published:** 2025-04-27

**Authors:** Philipp E. Chetverikov, Lourdes E. Peralta Alba

**Affiliations:** 1 Zoological Institute of Russian Academy of Sciences, Universitetskaya nab., 1, St. Petersburg 199034, Russia; 2 Laboratorio Entomologia y Acarologia Region del Maule, Servicio de Agricultura, Carmen 560, 3e piso, Curicó 3340001, Chile; lourdes.peralta@sag.gob.cl

**Keywords:** relict, endemic, life cycle, fruit pest, phytophagy, DNA barcoding

## Abstract

Acariform mites of the superfamily Eriophyoidea are permanent parasites of higher vascular plants. Seasonal morphological dimorphism in females has been documented across various eriophyoid taxa, while male dimorphism remains poorly understood. In this study, we analyzed morphological, molecular, and biological data from the genus *Austracus* Keifer 1944, with a particular focus on the type species, *A. havrylenkonis* Keifer 1944, associated with *Nothofagus*. Using new material collected from Chile and Argentina, we demonstrated that this species exhibits two distinct forms of both males and females, making it the first known bisexually dimorphic taxon within the family Phytoptidae. The summer form of *A. havrylenkonis* displays the unstable annulation of the dorsal opisthosoma, characterized by a significant variation in the number of thin, microtuberculated dorsal annuli interspersed among the broader, plate-like annuli typical of the winter form. This finding aligns with the previous observations of atypical deuterogyny in Eriophyoidea and leads us to hypothesize that gall mites employ diverse adaptive strategies—manifesting as either gradual or discrete morphological changes—to cope with seasonal environmental fluctuations. Investigating the genetic mechanisms underlying these adaptive strategies, along with further studies of eriophyoids associated with *Nothofagus* in the Southern Hemisphere, represents a promising direction for future research.

## Simple Summary

Gall mites (superfamily Eriophyoidea) are microscopic phytoparasites that transmit plant viruses and induce various abnormal growths, significantly affecting plant health and agricultural productivity. While most species within the Eriophyoidea superfamily are believed to have a simple life cycle, those with a complex life cycle often display seasonal dimorphism, characterized by two distinct female morphotypes: protogynes and deutogynes. In contrast, male dimorphism remains poorly understood due to the scarcity of males in populations and the historical focus on female morphology in Eriophyoidea systematics. *Trisetacus kirghisorum* (Nalepellidae), a conifer-associated species, is the only known eriophyoid taxon to exhibit seasonal dimorphism in both males and females. In this study, we analyzed morphological, molecular, and biological data from *Austracus havrylenkonis*, a gall mite species associated with Gondwanian relict host plants of the genus *Nothofagus*. Using material collected from Chile and Argentina, we demonstrated that this species exhibits two distinct forms of both males and females, marking it as the first known bisexually dimorphic taxon within the family Phytoptidae. We further discuss the gradual and discrete seasonal morphological changes observed in Eriophyoidea populations and emphasize the importance of additional studies on life cycles and faunistic surveys of gall mites associated with *Nothofagus* in Australasia.

## 1. Introduction

Eriophyoid mites (Acariformes, Eriophyoidea) are an ancient group of microscopic chelicerates highly specialized for phytoparasitism [1]. Recent taxa of eriophyoids are permanently associated with three large groups of vascular plants (ferns, conifers, and angiosperms); although, in previous epochs, eriophyoids or their ancestors may have inhabited various extinct groups of land plants, including seed ferns, the extinct lineages of gymnosperms, and flowering plants [2,3]. Current consensus between morphological systematics and molecular phylogenetics implies that the superfamily Eriophyoidea comprises four distinct groups—Pentasetacidae, Phytoptidae s.str., Nalepellidae and Eriophyidae s.l.—the last including members of two families, Eriophyidae and Diptilomiopidae [4]. Among these groups, only Phytoptidae s.str. is restricted to angiosperms, with a distinct tendency for associations with endemic and relict hosts [5].

Current data on the biology of phytoptids suggest they lack pronounced seasonal dimorphism, since morphologically distinct seasonal forms have never been reported in Phytoptidae s.str. A remarkable phytoptid taxon, the hazelnut bud mite *Phytoptus avellanae* s.l. Nalepa from *Corylus* spp., has an atypical life cycle, including a morphologically aberrant *Tegonotus*-like form of nymphs [6,7,8]. These nymphs are strikingly different morphologically from the adults in the way typically observed between (a) vagrant and concealed ecological groups of gall mites as well as between (b) seasonal forms of females in the complex life cycle of some species from the family Eriophyidae Nalepa [9]. A recent molecular phylogenetic study inferred two phytoptid species, *Austracus havrylenkonis* Keifer 1944 and *Sierraphytoptus alnivagrans* Keifer 1939, to be the possible sister taxa of *P. avellanae* [5]. Unlike *P. avellanae* that is a worldwide pest [7,10,11,12], both *A. havrylenkonis* and *S. alnivagrans* are rarely encountered species, putatively restricted to local areas in southern part of South America and in North America (USA: CA, WV), respectively, where their host plants (*Nothofagus* spp. and *Alnus* spp.) are naturally distributed.

For a decade, we attempted to obtain material from *Nothofagus* in order to reinvestigate poorly studied *Austracus* mites. In 2017, we received ethanol material from San Martín de los Andes (Argentina) containing branches of *Nothofagus antarctica* with fruits infested by *Austracus havrylenkonis* Keifer 1944. Later, we investigated two DNA isolates (F234 and d96) of *Austracus* from this material and obtained sequences of the 28S gene. Morphologically, the mite specimens corresponding to different isolates were similar; however, their 28S sequences were not identical, and species delimitation analysis suggested possible cryptic species [5].

In this paper, we aimed to summarize morphological, molecular, and biological data on the genus *Austracus* and reinvestigate the type species of this genus, *A. havrylenkonis* Keifer 1944, based on the previously studied material from San Martín de los Andes [5]. Surprisingly, when we carefully examined this material, we found that two distinct morphotypes of *Austracus* were present within one infested fruit of *Nothofagus*. In order to test the conspecificity of these morphotypes, we sequenced three marker genes and performed molecular phylogenetic analysis. This led to the unexpected discovery of the first documented case of pronounced bisexual dimorphism in Phytoptidae s.str. and highlighted the need to revise the generic diagnosis of *Austracus.*

## 2. Materials and Methods

### 2.1. Collection and Morphological Measurements

**Figure 1:**
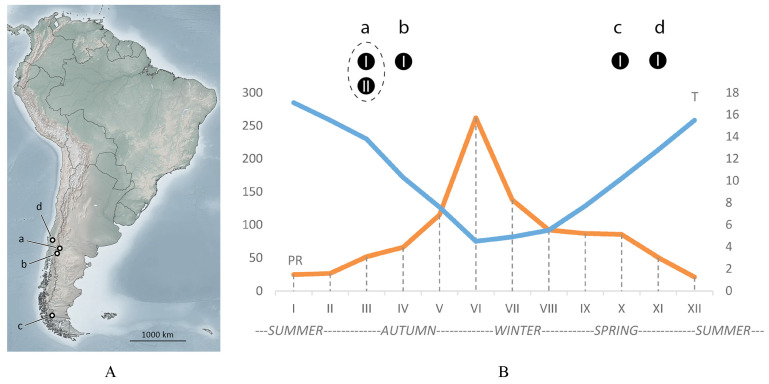
Four localities (a, b, c, d) of *Austracus* mites in Argentina and Chile (**A**) mapped on a seasonal climate chart of San Martín de los Andes (**B**), where the main material for this study was collected. The two curves are daily mean temperature (T °C, blue) and average precipitation (PR, mm, orange), both reconstructed based on data from https://www.smn.gob.ar/estadisticas (accessed on 25 January 2025). Black circles and letters a, b, c, d in (**B**) correspond to findings of *Austracus* in the four localities (a, b, c, and d) shown in (**A**); I and II—two morphotypes of *Austracus* (see the Section 3 for details).

Up to now, *Austracus* mites are known from four localities (a, b, c, d) in Argentina and Chile (Figure 1A, Table 1). This study is based mainly on the new material of *A. havrylenkonis* Keifer 1944 from the locality (a) near San Martín de los Andes (Argentina). This place is situated very close to the type locality (b) of *A. havrylenkonis* on the island Victoria in Nahuel Huapi National Park (Argentina), where in April 1943, this species was found for the first time inside the fruits of *Nothofagus dombeyi* [13]. For more than half of a century, no new records of this species were published. Recently, solitary females of *Austracus* cf *havrylenkonis* were found twice in southern Chile in region XII Magallanes (c): as accidental on a swamp sedge on 15 November 2015 in Laguna Parrillar National Reserve and as vagrant on very young leaves of sprouting *N. antarctica* on 15 November 2019 in Puerto Natales (Table 1). According to the official reports of Servicio Agricola y Ganadero (SAG) of the Ministry of Agriculture of Chile, mites of the genus *Austracus* have also been reported multiple times between 2016–2022 from central Chile (regions Araucania and Bio Bio); however, these reports did not include species-level identifications or confirm host associations, and the original material is unavailable for examination. We used the material from locality (a) for investigating morphology of *A. havrylenkonis* and the specimens from the locality (c) as additional material for comparison.

The branches of *N. antarctica* (Forster) Oerst. with maturing fruits were collected on 23 March 2017 by the late Dr. Vladimir Žikić (Serbia) in San Martín de los Andes, Argentina (Figure 1A, a) and kept in a vial with 70% ethanol in a freezer (−20 °C). In 2018, the ethanol material was transferred to Saint Petersburg (Russia) for investigation. The fruits were dissected with a sterile blade, and mites were collected by a needle and slide-mounted in modified Hoyer medium [22]. The external morphology of the slide-mounted specimens was studied using conventional light microscopy (LM) using a Leica DM2500 (Leica Microsystems GmbH, Wetzlar, Germany). Morphological descriptions were based on phase contrast (PC) and differential interference contrast (DIC) LM observations. All measurements were obtained using ToupTek ToupView software x64 v. 4.11.19728.20211022 (Hangzhou ToupTek Photonics Co., Hangzhou 310030, China). The terminology of eriophyoid morphology and the classification of Eriophyoidea follow [23] and [24], respectively. The drawings of mites were sketched by pencil using a video projector [25], scanned, and finalized in Adobe Illustrator CC 2014 using a Wacom Intuos S (CTL-4100K-N) graphics tablet (Wacom Co., Ltd, Kazo, Saitama, Japan).

### 2.2. DNA Extraction and Sequencing

For DNA extraction, 1–2 females of morphotypes I and II, distinguished by coloration, were separately crushed with a fine pin in a 1.5 μL drop of distilled water on a cavity well microscope slide. The fragments of the posterior part of the mite bodies (including morphotype specific dorsal opisthosomal annuli) were pulled out of the drop and slide-mounted for morphotype confirmation. Each drop was pipetted into a thin-walled PCR tube with 30 μL of 6% solution of Chelex^®^ 100 Resin Bio Rad before being heated three times (5 min at 95 °C) in a thermostat with intermediate short vortexing. The solution above the Chelex^®^ granules was used as the DNA template for PCR to amplify the D1-D2 domains of the 28S rDNA, ITS1-5.8S-ITS2, and mitochondrial Cox1 genes. For the PCR and sequencing, we applied the protocols and primers detailed by [26]. Sequences were obtained using BigDye Terminator v.3.1 chemistry (Applied Biosystems, Foster City, CA, USA) and a 3500xl Genetic Analyzer (Applied Biosystems). Trace files were checked and edited using GeneStudioTM Professional 2.2.0.0 (www.genestudio.com, accessed on 20 May 2019). Overall, we obtained nine DNA isolates, six Cox1 sequences, eight ITS1-5.8S-ITS2 sequences, and four D1D2 28S sequences of *A. havrylenkonis* (Table 2).

### 2.3. Sequence Alignment and Molecular Phylogenetic Analyses

Combined molecular phylogenetic analyses of Cox1 and D1D2 28S sequences were carried out to test the conspecificity of the morphotypes I and II of *A. havrylenkonis*. The ITS1–5.8S–ITS2 sequences were used only for intra-genus comparisons among *Austracus* DNA isolates. Sequences of 28S and Cox1 genes of *Fragariocoptes setiger* (JAIFTH010000001.1, JAIFTH010000002.1) and *Phytoptus lineatus* (MT712456, MT712746) were used as distant and close out-groups, respectively. We combined them with 28S and Cox1 sequences of *Austracus* listed in Table 2 and obtained the dataset for analyses. All sequences were aligned with the MAFFT algorithm [27] through the web-based program interface [28] using default settings. Maximum likelihood analyses were conducted in IQ-tree 2 [29]. For 28S/Cox1 gene evolution, the HKY+F+I/TPM2u+F+I models were selected using ModelFinder [30] as implemented in IQ-tree 2 based on the Bayesian information criterion. Branch support values were generated from Ultrafast bootstrap approximation (UFBoot) with 10,000 bootstrap alignments, 1000 maximum iterations, and a minimum correlation coefficient of 0.99. Values of a single branch test (SH-like approximate likelihood ratio test (SH-aLRT) with 1000 replicates [31]) were labeled on the maximum likelihood (ML) trees.

## 3. Results

### 3.1. Microscopic Observations and Morphotypes of Austracus havrylenkonis

**Figure 2:**
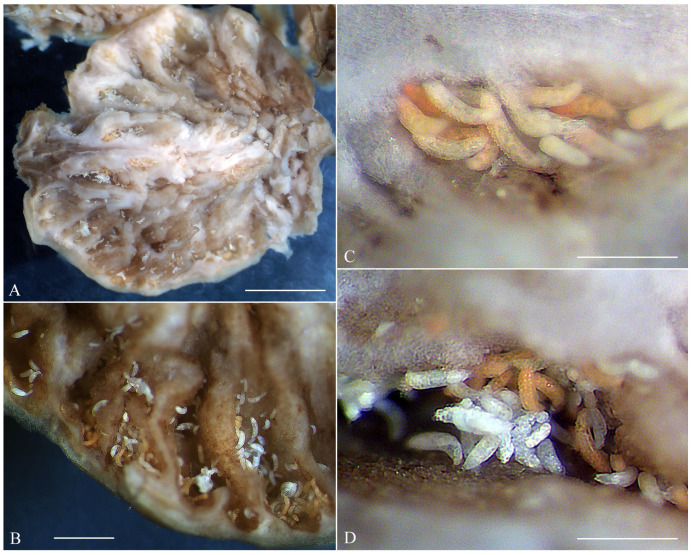
Dissected fruit of *Nothofagus antarctica* infested by *Austracus havrylenkonis* Keifer 1944. (**A**,**B**)—fragments of the dissected fruit with numerous mites; (**C**)—cluster of light and bright orange mites (mostly of the morphotype I); (**D**)—mixed group of mites of the morphotypes I and II. Scale bar: (**A**)—25 mm, (**B**)—1 mm, (**C**,**D**)—300 μm.

The fruits of *Nothofagus antarctica* infested by *A. havrylenkonis* are swollen and globose. Numerous mites, ranging in color from white to bright or light orange, were observed clustered in groups between folds of the internal fruit tissues (Figure 2). Under a stereomicroscope, some of the white-colored adult mites appeared notably larger, being both longer and thicker, while most (though not all) of the orange mites were slightly shorter and slender. Immatures were significantly less numerous than adults, and all exhibited a whitish or semi-translucent appearance. Microscopic observations of slide-mounted adult mites revealed two morphotypes (MT-I and MT-II, see below) in both females and males. While these morphotypes were not perfectly correlated with color, all larger white mites corresponded to morphotype I, and all smaller bright orange mites belonged to morphotype II.

#### 3.1.1. Female and Male Morphotype I (MT-I)

**Figure 3:**
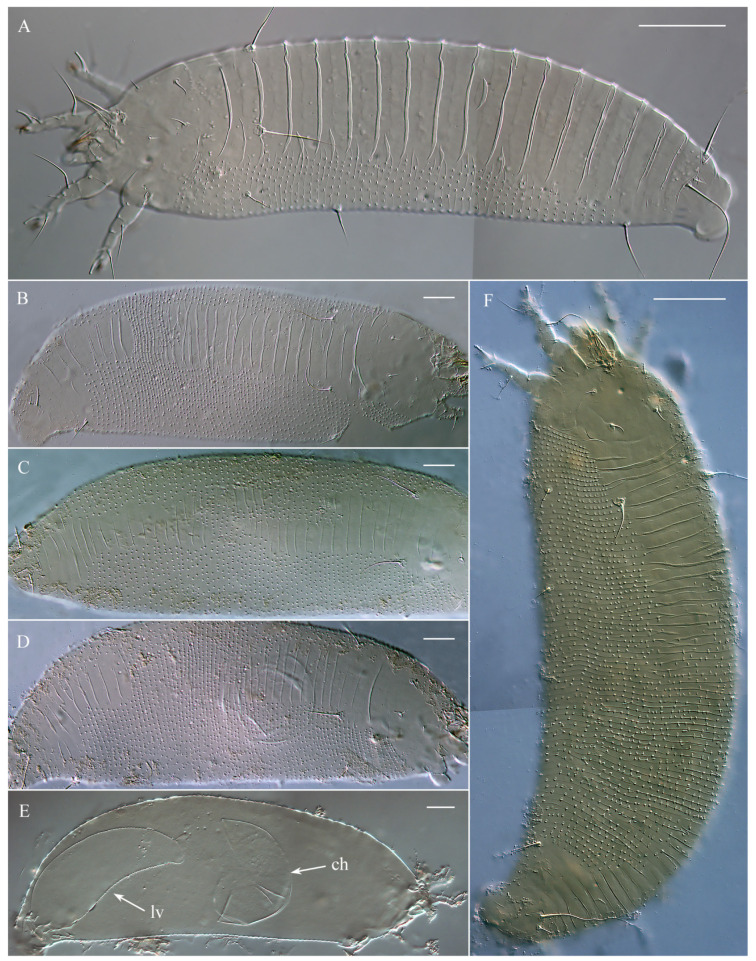
DIC LM microphotographs of morphotypes MT-I (**A**) and MT-II (**B**–**F**) of *Austracus havrylenkonis* Keifer 1944 (females) from fruits of *Nothofagus antarctica* from San Martín de los Andes (Argentina). Note: ruptured chorion (ch) and larva (lv) inside a female of morphotype II are shown in (**E**). Scale bar: (**A**,**F**)—40 μm; (**B**–**E**)—20 μm.

These specimens are morphologically similar to those described by Keifer [13] from locality *b* and later recorded in localities *c* and *d* (Figure 1A). They exhibit a light-to-bright orange coloration, with smooth, broad, and slightly overlapping plate-like dorsal annuli, as well as microtuberculated narrow ventral annuli that are significantly more numerous than the dorsal ones (Figure 3A).

#### 3.1.2. Female and Male Morphotype II (MT-II)

This new form of *Austracus* was found for the first time in this study in locality ***a*** (Figure 1A). These mites were predominantly white, with some individuals displaying a light orange coloration. They had irregular annulation of the dorsal opisthosoma, which shows a wide range of shapes from minimal differentiation of the dorsal opisthosomal annuli into broader and narrower annuli to distinctly heterogeneous patterns, where some dorsal annuli are as thin and microtuberculated as the ventral ones, while others are significantly wider and smooth (Figure 3B–D,F).

### 3.2. Molecular Phylogenetics

To test the genetic identity of the morphotypes I and II, we obtained sequences of three marker genes—Cox1, ITS, and D1D2 28S (Table 2). The Cox1 and ITS sequences from the mites of different morphotypes were identical. D1D2 28S sequences were almost identical except one nucleotide position in the middle of D2 region of 28S gene: “G” in isolates d488 (MT-I), d492 (MT-II), and d493 (MT-II) vs. “A” in isolate d490 (MT-I). This mononucleotide variation in the 28S gene does not correlate with mite morphotype. Overall, a sequence comparison indicates that the morphotypes I and II are conspecific.

Only one Cox1 sequence, two 28S sequences, and no ITS sequences of *Austracus* are present in GenBank (accessed on 25 February 2025). Nucleotide blast (BLASTN) for our longest Cox1 sequence (PQ406503, isolate d488) revealed three Cox1 sequences of phytoptids to be the closest: MT712721 (*A. havrylenkonis* isolate F234, 86.9% identity, 100% coverage), MT712756 (*Solenocristus searsius*, 86.3% identity, 100% coverage), and MT712739 (*Phytoptus chamaebatiae*, 86.4% identity, 99% coverage). Calculated Kimura two parameter (K2P) genetic distance between sequences PQ406503 and MT712721 of *A. havrylenkonis* is equal to 16% ± 2%.

Protein blast (BLASTX) for the same Cox1 sequence (PQ406503) revealed one 100% identical sequence (QLD94595 of *A. havrylenkonis* isolate F234) and two sequences with 97–98% identity (ATY50385 of Novophytoptus rostratae and QLD94620 of *Phytoptus lineatus*), all of them with 99% coverage. The 100% identity of amino acid sequences PQ406503 and QLD94595 indicates that the differences between the Cox1 nucleotide sequences of *Austracus* from our material (PQ406503) and the sequence of *Austracus* isolate F234 from GenBank (MT712721) are due to synonymous substitutions.

Blast search for our four new 28S sequences of *Austracus* mites of different morphotypes revealed the three most similar sequences of phytoptids with 99–100% coverage: MT712437 (*Austracus* sp. isolate d96, 99.9–100% identity), MT712438 (*A. havrylenkonis* isolate F234, 99.0–99.1% identity), and KT070303 (*Sierraphytoptus alnivagrans*, 93.9–94.1% identity).

**Figure 4:**
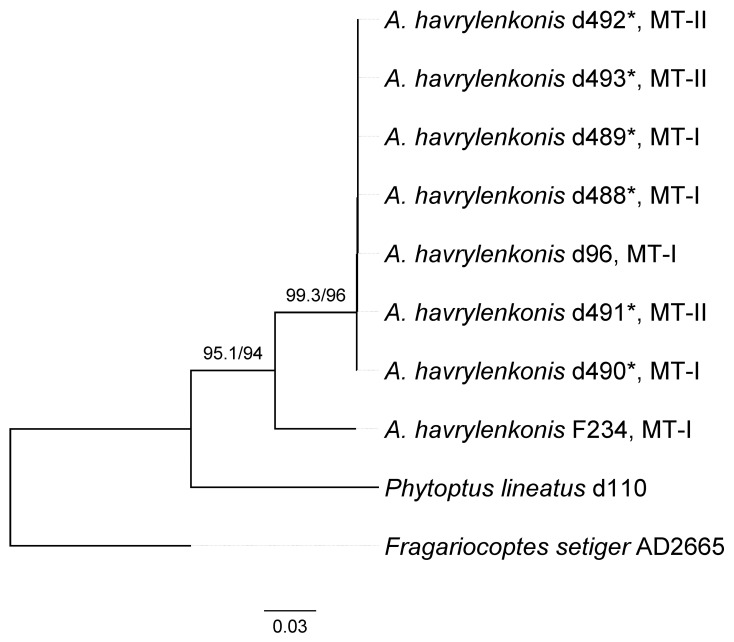
Combined Cox1 + D1D2 28S ML tree of eight isolates of *Austracus havrylenkonis* Keifer 1944. Values of SH-aLRT /UF support are indicated above branches. MT-I and MT-II—morphological morphotypes I and II. Asterisks indicate new isolates obtained in this study (Table 2).

A combined (Cox1 + D1D2 28S) molecular phylogenetic analysis revealed monophyletic *Austracus* diverging into two clades, one including a single isolate F234 from GenBank and the other comprising all the other isolates (Figure 4). Isolates representing different morphotypes do not form monophyletic groups, indicating that MT-I and MT-II are two morphological forms but not different species.

### 3.3. Seasonal Findings of Austracus and Data on Molting, Sperm Storage, and Putative Dispersal

Seasonal changes in temperature and precipitation in San Martín de los Andes are shown in Figure 1B. In this area, winter (May–July) is cold and wet, but summer (December–February) is warm and dry. In Argentina (localities *a* and *b*, Figure 1A), *Austracus* mites were found in autumn. Findings in Chile in localities *c* and *d* were made at the end of spring.

Morphotype MT-I was recorded in all four localities (*a*, *b*, *c*, *d*) inside fruits (*a*, *b*) and as vagrant (*c*, *d*), whereas morphotype MT-II was found only in fruits in the locality (*a*).

In the mixed population from San Martín de los Andes (autumn sample), females of the morphotype MT-II often contained developing eggs and larvae eclosing inside (Figure 3D,E), which is a clear indication of female senility [32]. In the spermathecae of most partially cleared MT-I females from that sample, we observed clusters of sperm cells; however, no MT-I females with developing eggs were found, suggesting no reproduction in that period.

Remarkably, those were MT-I females that were detected in early spring in the Chilean samples from Magallanes (Figure 1A, *c*) as vagrants and as accidental on atypical host, probably in the period of dispersal after overwintering in fruits.

Overall, our limited seasonal data combined with the results of the sequence comparison and molecular phylogenetic analyses (reported above) indicate conspecificity of MT-I and MT-II and suggest that MT-II is a summer form (protogyne) and MT-I is a winter form (deutogyne) of *A. havrylenkonis*. The form MT-I was described by Keifer [13], and MT-II is a newly discovered seasonal form.

### 3.4. Morphological Differences Between Seasonal Morphotypes of Austracus havrylenkonis

**Figure 5:**
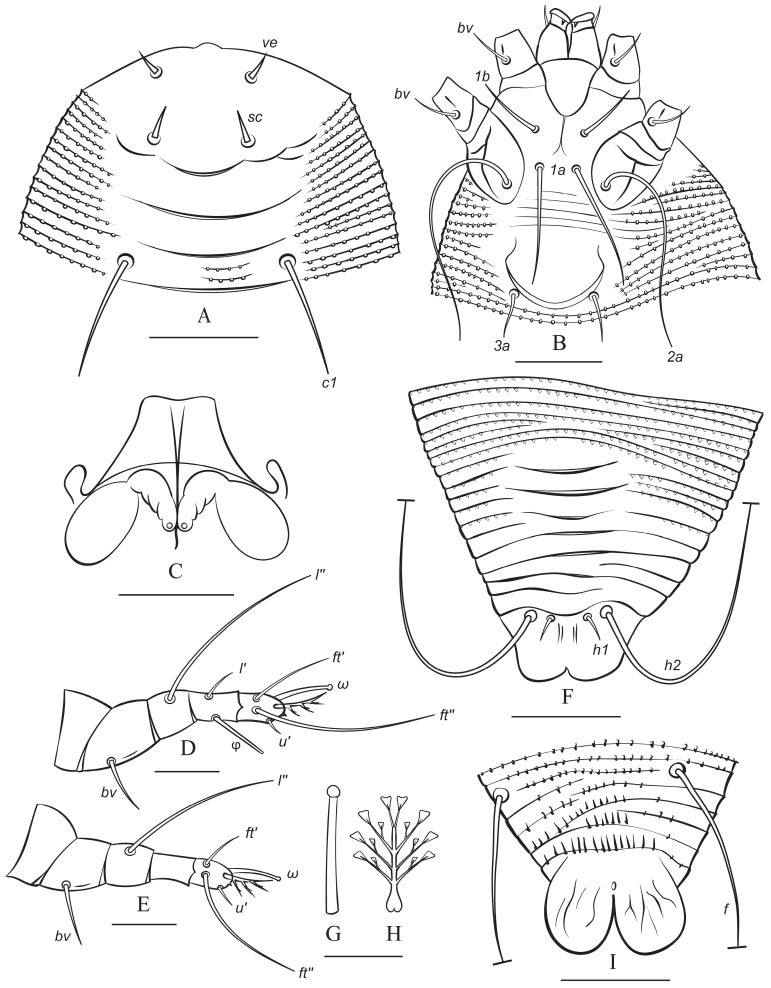
Drawings of protogyne female (summer form) of *Austracus havrylenkonis* Keifer 1944. (**A**)—prodorsal shield and dorsal view of anterior part of opisthosoma; (**B**)—coxigenital area; (**C**)—internal genitalia; (**D**,**E**)—legs I and II; (**F**)—dorsal view of rear part of opisthosoma; (**G**,**H**)—tarsal solenidion I (**G**) and tarsal empodium I (**H**); (**I**)—ventral view of telosoma. Scale bar: (**A**,**F**,**I**)—30 µm; (**B**)—25 µm; (**C**)—1 µm 5; (**D**,**E**)—10 µm; (**G**,**H**)—5 µm.

**Figure 6:**
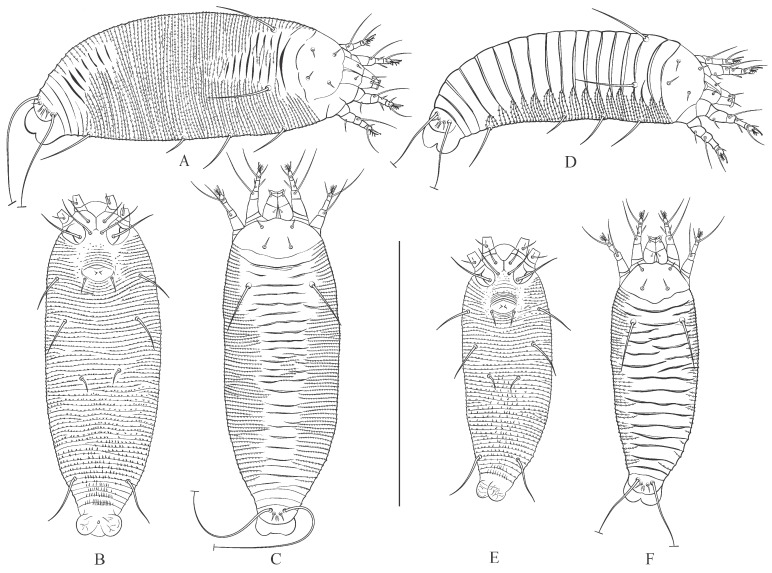
Drawings of summer (**A**–**C**) and winter (**D**–**F**) forms of females (**A**,**D**) and males (**B**,**C**,**E**,**F**) of *Austracus havrylenkonis* Keifer 1944. Scale bar: (**A**–**F**)—250 µm.

The summer and winter forms of *A. havrylenkonis* are very similar in most morphometric characters, except for the dimensions of the body and prodorsal shield, distances between prodorsal shield setae, and the number of opisthosomal annuli (Table 3). Although the values of all these morphometrics exhibit marginal overlap, the summer forms of both sexes generally display a longer and broader body, a wider prodorsal shield, greater pairwise distances between setae *ve* and *sc*, and a higher number of opisthosomal annuli compared to the winter forms. The topography of the dorsal opisthosoma serves as the most distinctive qualitative trait differentiating these forms (Figure 3, Figure 5 and Figure 6). In the winter form (MT-I), both females and males exhibit a slightly flattened dorsal opisthosoma, characterized by broad, partially overlapping plate-like dorsal annuli. In contrast, the summer form (MT-II) of both sexes also displays plate-like annuli, but these are notably narrower and are often interrupted in the mid-region of the opisthosoma by thinner, microtuberculated annuli. These thin annuli in summer forms vary significantly in number, resulting in a continuum of numerous intermediate forms (Figure 3B–D,F).

## 4. Discussion

**Seasonal dimorphism in populations of Eriophyoidea: gradual vs. discrete morphological changes.** Seasonal morphological dimorphism in the females of Eriophyoidea was first reported by Putman [33]. Subsequently, this phenomenon was documented by Keifer [34], who coined the term “deuterogeny”, designating summer females as “protogynes” and winter females as “deutogynes”. In this study, we show that *Austracus havrylenkonis* Keifer 1944, which inhabits fruits of *Nothofagus* spp., has a complex life cycle with seasonal dimorphism present in both sexes, making it the first bisexually dimorphic species associated with an angiosperm host within the family Phytoptidae s. str. Until now, seasonal dimorphism in both sexes has only been documented in *Trisetacus kirghisorum* Shevchenko 1962 (Nalepellidae), infesting the seeds of junipers [35]. Males of eriophyoid mites have received limited attention from acarologists for two primary reasons: (a) males are typically far less abundant than females in populations, and (b) male morphology offers limited diagnostic value, as the systematics of Eriophyoidea has historically relied on female morphological traits. While male dimorphism remains poorly understood across most eriophyoid genera, further research into the life cycles of gall mites from diverse taxa is necessary to determine whether bisexual seasonal dimorphism represents a lineage-specific homoplastic adaptation or a symplesiomorphic trait of Eriophyoidea.

There is significant variation in the extent to which seasonal forms differ morphologically across various eriophyoid taxa [36]. In some cases, the differences are nearly imperceptible, while in others, the seasonal forms may be so morphologically distinct that they could be classified as separate species or even genera. Seasonal dimorphism in *Austracus* is particularly notable, as the summer form exhibits the unstable annulation of the dorsal opisthosoma. There is considerable variation in the number of thin, microtuberculated dorsal annuli interspersed between the broader plate-like annuli (Figure 3B–D,F), which are characteristic of the winter form (Figure 3A). Interestingly, Keifer [37] (p. 2) observed that, in a population of the North American species *Aculops rhoicecis* (Eriophyidae) from leaf galls of *Rhus trilobata* Nutt. (Anacardiaceae), some females with “intermediate characteristics” were present alongside morphologically distinct protogynes and deutogynes. A somewhat similar case was reported in *Oziella atherodes* Chetverikov, 2011 (Phytoptidae s.str.), found inside leaf sheaths of the sedge *Carex atherodes* Spreng. (Cyperaceae) in Northwestern Russia. Alongside larger protogynes with a higher number of empodial rays and smaller deutogynes with fewer empodial rays, there was a series of differently sized females with intermediate numbers of empodial rays representing a gradual morphological transition from protogynes to deutogynes [38]. In contrast to these cases (corresponding to “atypical deuterogeny” sensu Manson and Oldfield [36]), dimorphism in some species of Eriophyoidea is distinctly non-gradual but discrete (“typical deuterogeny”). For example, in the genus *Shevchenkella*, protogynes and deutogynes exhibit entirely distinct morphologies with no intermediate forms [34,39]. We hypothesize that these “gradual” and “discrete” seasonal morphological differences reflect different adaptive strategies of gall mites to their environment. Investigating the genetic mechanisms underlying these strategies represents a compelling direction for future research.

**Is *Austracus havrylenkonis* a complex of cryptic species**? Cryptic speciation is a widespread evolutionary phenomenon observed across diverse groups of living organisms, including arthropods, amphibians, fish, and plants [40,41]. Among these, mites (Acari) represent a particularly notable example, as their high morphological uniformity and small size often obscure significant genetic divergence, leading to the discovery of numerous cryptic species within traditionally recognized taxa [42,43]. Because of the unique ecological traits and population structures of Eriophyoidea (as discussed by Sabelis and Bruin [44]), cryptic speciation is considered especially common in this group of mites [43]. Among the most striking examples are species complexes within the genera *Aceria* and *Abacarus* from herbaceous monocots extensively studied in the first decades of the XXI century [45,46,47,48]. Some grass-inhabiting species in these genera show significant genetic divergence (up to ~20% in Cox1 sequences), yet they are so morphologically similar that they can only be differentiated through comprehensive multivariate statistical analyses [43].

In this study, we encountered an intriguing case where nucleotide sequences of the Cox1 gene from specimens of *A. havrylenkonis* from different fruits of the same host-plant individual showed significant differences (K2P = 16%; PQ406503, isolate d488 vs MT712721, isolate F234). Remarkably, all these differences represented synonymous substitutions, resulting in no changes to the amino acid sequences. This pattern may reflect the clonal structure of *A. havrylenkonis* and the isolated evolution of its populations within the fruits, where genetic differences accumulate due to the prolonged absence of gene flow, despite the likely absence of reproductive barriers. On the other hand, biogeographic data on the host plants of *Austracus* suggest an alternative scenario. The genus *Nothofagus* is an ancient Gondwanan plant lineage, now distributed in Australasia and South America [49,50]. A series of ten vicariant species of *Nothofagus* is distributed across a vast area of central and southern Chile, as well as in Argentina [51], the regions characterized by diverse geographical barriers and climatic conditions [52,53,54]. As a result, the formation of host-specific races and complexes of genetically diverging species of gall mites associated with *Nothofagus* is highly probable. This question could be addressed through a longitudinal screening in Chile, examining different South American species of *Nothofagus*. Additionally, it would be interesting to search for phytoptid mites in the eastern part of the Southern Hemisphere—specifically, in New Zealand, New Guinea, New Caledonia, and Australia—regions rich in endemic species of *Nothofagus*. Such a study could help determine whether *Austracus* or other phytoptid mites are present in these regions, how they have evolved morphologically compared to South American phytoptids associated with *Nothofagus*, and what genetic differences they have acquired over the long period of isolation.

**The problematic diagnosis of the genus *Austracus*.** Keifer [13] described the monotypic genus *Austracus* based on females showing deutogyne characters, which contradicts current tradition to compose a generic diagnosis based on morphology of protogyne females [24]. Based on our new morphological data on summer females of *A. havrylenkonis*, the diagnosis of *Austracus* should incorporate the distinctive opisthosomal features of protogynes—specifically, the presence of two types of dorsal annuli: broad plate-like annuli interspersed with thin, microtuberculated annuli. However, this proposed amendment requires further validation, as undescribed *Austracus* species may exhibit protogyne opisthosomal morphology that differs from the pattern observed in *A. havrylenkonis*.

Among other phytoptid taxa, the protogynes of *A. havrylenkonis* are closest to the species of the group I of the genus *Phytoptus* Dujardin 1851 (Phytoptidae, Phytoptinae) that retain *φ* I. Contrary to *Phytoptus* characterized by opisthosomal annuli that are not differentiated dorso-ventrally, protogynes of *A. havrylenkonis* have several dorsal plates (wider opisthosomal annuli) on their dorsal opisthosoma along with the typical narrow dorsal opisthosomal annuli.

Deutogynes of *A. havrylenkonis* are very close to a phytoptid genus *Solenocristus* Chetverikov et al., 2018 (tribe Sierraphytoptini). They differ in the position of setae *ve* (displaced before the antero-lateral margin of prodorsal shield in *Solenocristus*) and the presence of a large subtriangular frontal lobe of prodorsal shield and middorsal opisthosomal ridge (both are absent in *A. havrylenkonis*). The deutogynes of *A. havrylenkonis* are also similar to the genera *Fragariocoptes* Roivainen 1951 and *Sierraphytoptus* Keifer 1939 (tribe Sierraphytoptini), but these two genera lack tibial solenidion *φ* I (present in *A. havrylenkonis*) and have tubercles of setae *ve* displaced forward and positioned just before the antero-lateral margin of prodorsal shield (*ve* not displaced in *A. havrylenkonis*). Finally, deutogynes of *A. havrylenkonis* also bear a distant resemblance with some vagrant phytoptids associated with palms, e.g., *Mackiella* Keifer 1939 and *Retracrus* Keifer 1965 (tribe Mackiellini), but these phytoptids lack opisthosomal setae *c1*, which are present in *A. havrylenkonis*.

## 5. Conclusions

Taxonomic research on Eriophyoidea is advancing rapidly, with numerous new species being described annually. Beyond these taxonomi developments, studies on the life cycles of Eriophyoidea, combined with DNA genotyping, are of critical importance, particularly for evaluating the conspecificity of morphologically dissimilar sympatric mite individuals that correspond to different morphotaxa [39,53]. Our research on *A. havrylenkonis* revealed that morphologically distinct seasonal forms of this species share identical sequences across three marker genes. We also identified *A. havrylenkonis* as the second documented case of a bisexually dimorphic taxon within Eriophyoidea, suggesting that seasonal dimorphism in males may be more widespread among gall mites than previously recognized. Additionally, we documented a new instance of atypical seasonal dimorphism in *A. havrylenkonis*, characterized by an unstable topography of the dorsal opisthosoma, which aligns with the previously described phenomenon of “atypical deuterogeny”. Given the labor-intensive nature of biological studies on Eriophyoidea, we are only beginning to uncover the diverse adaptive strategies these mites employ. A compelling question that warrants further investigation is why different sympatric Eriophyoidea taxa, despite coexisting under similar environmental conditions, exhibit varying degrees of seasonal morphological variation within their populations. This question could be explored in future research through the application of functional genomics, transcriptomics, and proteomics, which may provide deeper insights into the evolutionary and ecological dynamics of this aberrant group of ancient microscopic phytoparasites.

## Figures and Tables

**Table 1:** Published records of *Austracus* in Chile and Argentina. Asterisks (*) indicate the findings that are not shown in Figure 1 because of insufficient data.

Mite Species	Host	Collecting Data	Reference	Designation in Figure 1
*A. havrylenkonis* Keifer 1944	*N. dombeyi* (inside fruits)	April 1943, Nahuel Huapi National Park (Argentina)	[13]	(a)
*A. havrylenkonis* Keifer 1944	*N. antarctica* (inside fruits)	23 March 2017, San Martín de los Andes (Argentina)	[5]	(b)
*A. havrylenkonis* Keifer 1944	Accidental females on a swamp sedge	15 November 2015, Laguna Parrillar National Reserve, Magallanes (Chile)	[14]	(c)
*A. havrylenkonis* Keifer 1944	*N. antarctica* (vagrant females on young leaves)	15 November 2019, Puerto Natales, Ultima Esperanza, Magallanes (Chile)	[15]	
*A. havrylenkonis* Keifer 1944	*N. dombeyi*	4 October 2018, La Araucania, comuna Curarrehue, Villarica (Chile)	[16]	(d)
*Austracus* sp.	*N. dombeyi*	3 February 2020, La Araucania, comuna Curarrehue, Villarica (Chile)	[17]	*
*Austracus* sp.	*N. dombeyi*	2 September 2020, La Araucania, Vilcun (Chile)	[18]	*
*Austracus* sp.	*N. dombeyi*	8 June 2016, La Araucania, Vilcun (Chile)	[19]	*
*Austracus* sp.	*N. dombeyi*	7 September 2020, La Araucania, Angol (Chile)	[20]	*
*Austracus* sp.	*N. pumilio*	4 April 2016, Alto Bio Bio (Chile)	[21]	*

**Table 2:** Accession numbers of partial Cox1, ITS, and D1D2 28S sequences of mites *Austracus havrylenkonis* differing in color and external morphology (morphotypes I and II) from Argentina.

DNA Isolate	Morphotype (MT) and Number of Mites Used for DNA Extraction	Mite Color	Cox1	ITS1-5.8S-ITS2	D1D2 28S	Reference
d488	MT-I (*n* = 1)	orange	PQ406503 (510 bp)	PQ421013 (652 bp)	PQ421009 (971 bp)	this study
d489	MT-I (*n* = 1)	orange	PQ406504 (492 bp)	PQ421014 (703 bp)	–	this study
d490	MT-I (*n* = 2)	orange	PQ406505 (502 bp)	PQ421015 (703 bp)	PQ421010 (976 bp)	this study
d491	MT-II (*n* = 1)	white	PQ406506 (502 bp)	PQ421016 (643 bp)	–	this study
d492	MT-II (*n* = 1)	white	PQ406507 (399 bp)	PQ421017 (660 bp)	PQ421011 (975 bp)	this study
d493	MT-II (*n* = 2)	white	PQ406508 (445 bp)	–	PQ421012 (970 bp)	this study
d496	MT-I (*n* = 1)	orange	–	PQ421018 (432 bp)	–	this study
d497	MT-II (*n* = 1)	white	–	PQ421019 (614 bp)	–	this study
d499	MT-II (*n* = 1)	white	–	PQ421020 (674 bp)	–	this study
F234	MT-I (*n* = 1)	white	MT712721	–	MT712438	[5]
d96	MT-I (*n* = 1)	white	–	–	MT712437	[5]

**Table 3:** Measurements (means followed by ranges) of summer and winter forms of *Austracus havrylenkonis* Keifer 1944. Characters that distinguish between forms are highlighted in gray. Remarks: only numbers of wider plate-like dorsal annuli are given for summer females and males, whereas highly variable numbers of narrower microtuberculated dorsal annuli situated between them are not given.

Characters	Summer Females (MT-II), n = 11	Winter Females (MT-I), n = 12	Summer Males (MT-II), n = 7	Winter Males (MT-I), n = 8
Length of body	340 (295–388)	308 (283–328)	309 (282–350)	226 (210–250)
Width of body	107 (98–119)	91 (86–96)	106 (99–114)	90 (84–100)
Length of prodorsal shield	44 (41–53)	42 (39–46)	47 (44–50)	45 (41–50)
Width of prodorsal shield	75 (70–82)	60 (58–74)	84 (80–91)	72 (67–78)
Length of *ve*	5 (5–6)	7 (5–8)	6 (5–7)	7 (6–7)
Distance between *ve*	29 (26–32)	23 (20–27)	35 (34–35)	29 (27–31)
Length of *sc*	7 (6–9)	10 (8–11)	8 (6.5–9)	9 (8–9)
Distance between *sc*	27 (25–30)	23 (20–24)	31 (30–31)	27 (25–30)
Distance between *ve* and *sc*	20 (18–22)	20 (18–21)	21 (20–21)	19 (17–21)
Length of gnathosoma	26 (24–29)	27 (24–30)	28 (27–28)	26 (23–30)
Length of suboral plate	16 (15–17)	17 (14–18)	17 (16–17)	16 (15–17)
Width of suboral plate	16 (15–17)	17 (14–20)	19 (18–20)	16 (15–18)
Length of chelicera	22 (19–24)	23 (21–24)	21 (19–23)	21 (18–22)
Length of *d* (s. antapic)	3 (2–4)	4 (2–5)	4 (3–5)	3 (2–4)
Length of *v*	2 (1–3)	2 (1–2)	2 (1–2)	1 (0.5–2)
Length of *ep*	2 (2–4)	3 (2.5–4)	3 (2–3)	3 (2–4)
Length of leg I	39 (37–42)	40 (39–43)	38 (37–38)	38 (37–39)
Length of tarsus I	8 (7–9)	9 (8–10)	8 (7–8)	8 (6–10)
Length of *ω* I	10 (9–13)	11 (10–12)	8 (6–9)	9 (8–10)
Length of empodium I	7 (6–8)	7 (6–8)	8 (7–8)	7 (6.5–8)
Number of rays of empodium I	4 (4–4)	4 (4–4)	4 (4–4)	4 (4–4)
Length of *ft*’ I	9 (7–14)	15 (10–17)	12 (10–13)	11 (8–14)
Length of *ft*” I	28 (23–32)	33 (30–37)	30 (29–30)	24 (19–32)
Length of *u*’ I	4 (2–5)	4 (3.5–5)	5 (4–5)	4 (2.5–5)
Length of tibia I	8 (7–9)	9 (8–11)	8 (7–8)	7 (5–8)
Length of ϕ	11 (10–12)	11 (10–12)	11 (10–12)	11 (9.5–12)
Length of *l*’ I	4 (3–5)	6 (5–7)	5 (4–6)	4 (3–5)
Length of genu I	7 (7–8)	9 (8–10)	7 (6–7)	7 (6–8)
Length of *l*’’ I	31 (26–34)	30 (27–33)	33 (32–34)	29 (27–32.5)
Length of femur I	12 (11–13)	12 (11–13)	12 (11–12)	11 (9.5–12)
Length of *bv* II	9 (7–10)	9 (7–13)	9 (8–10)	10 (7–13)
Length of leg II	37 (35–39)	39 (36–41)	37 (36–37)	35 (32–36)
Length of tarsus II	8 (7–9)	9 (8–10)	7 (6–7)	8 (6–9)
Length of *ω* II	9 (7–11)	12 (10–16)	10 (9–10)	9 (9–10)
Length of empodium II	6 (5–7)	6 (5–7.5)	8 (7–8)	7 (7–8)
Number of rays of empodium II	4 (4–4)	4 (4–4)	4 (4–4)	4 (4–4)
Length of *ft*’ II	6 (5–7)	7 (6–8)	4 (3–5)	5 (4–6)
Length of *ft*” II	25 (21–29)	30 (26–33)	26 (25–27)	27 (23–29)
Length of *u*’ II	4 (3–5)	5 (4–6)	5 (4–5)	4 (3–4)
Length of tibia II	6 (5–7)	8 (7–9)	7 (6–7)	5 (4.5–6)
Length of genu II	7 (6–8)	8 (7–9)	7 (6–7)	6 (5.5–7)
Length of *l*” II	26 (20–29)	25 (21–29)	29 (27–30)	27 (24–30)
Length of femur II	10 (7–12)	13 (11–13.5)	10 (9–11)	11 (10–12)
Length of *bv* II	12 (9–17)	12 (10–16)	14 (12–16)	11 (7–13)
Length of prosternal apodeme	7 (6–8)	8 (7–11)	9 (8–10)	8 (6–9)
Length of *1a*	26 (21–38)	27 (22–37)	27 (26–27)	27 (21–38)
Length of *1b*	14 (10–19)	13 (7–16)	14 (13–15)	12 (11–13)
Length of *2a*	46 (37–62)	50 (32–63)	40 (31–48)	39 (30–48)
Distance between *1a*	11 (10–13)	12 (10–14)	11 (10–12)	11 (10–11)
Distance between *1b*	17 (14–18)	15 (14–19)	16 (15–17)	15 (14.5–16)
Distance between *2a*	32 (29–35)	29 (26–34)	34 (32–35)	27 (24–29)
Length of *3a*	10 (8–12)	11 (10–13)	10 (9–11)	12 (10–13)
Distance between *3a*	26 (22–29)	23 (21–31)	26 (25–27)	23 (22–24)
Number of coxigenital annuli	5 (3–6)	5 (5–6)	7 (7–7)	5 (5–5)
Length of epigynium/male genital area	20 (19–23)	21 (19–24)	10 (9–13)	11 (9–14)
Width of epigyium/male genital area	28 (27–31)	29 (27–30)	27 (25–29)	25 (24–28)
Number of ventral annuli	72 (65–82)	63 (60–66)	60 (59–61)	50 (45–52)
Number of dorsal annuli	22 (12–33)	17 (16–18)	22 (18–29)	18 (16–19)
Length of *c1*	42 (36–54)	47 (36–59)	41 (34–48)	46 (41–50)
Length of *c2*	21 (14–26)	20 (17–24)	23 (19–27)	21 (17–25)
Length of *d*	24 (20–28)	20 (15–26)	27 (25–30)	20 (15–26)
Length of *e*	11 (8–13)	13 (10–17)	13 (12–14)	13 (11–15)
Length of *f*	30 (20–37)	37 (29–42)	38 (37–40)	31 (30–34)
Length of *h1*	7 (5–9)	7 (6–10)	6 (5–7.5)	6 (5–7)
Length of *h2*	73 (54–85)	101 (75–116)	78 (72–84)	68 (60–79)
Number of annuli before *c2*	12 (10–14)	10 (9–10)	11 (10–12)	9 (8–10)
Number of annuli between *c2* and *d*	15 (11–18)	13 (12–16)	10 (9–10)	10 (9–10)
Number of annuli between *d* and *e*	12 (11–14)	13 (10–15)	11 (10–12)	8 (6–9)
Number of annuli between *e* and *f*	28 (23–33)	23 (20–25)	24 (23–24)	19 (18–20)
Number of annuli between *f* and *h*	5 (4–6)	5 (4–6)	5 (4–6)	5 (4–6)

## Data Availability

All new DNA sequences obtained in this study have been deposited in the National Center for Biotechnology Information (NCBI) GenBank database (https://www.ncbi.nlm.nih.gov/genbank) (accessed on 25 March 2025).
